# Sorafenib priming may augment salvage chemotherapy in relapsed and refractory FLT3-ITD-positive acute myeloid leukemia

**DOI:** 10.1038/bcj.2014.59

**Published:** 2014-08-08

**Authors:** K D Cummins, S M Jane, S Nikovic, A Bazargan, R Filshie, G Sutrave, M Hertzberg, A Scott, S Lane, C K Yannakou, D Ritchie, J D'Rozario, J Black, K Bavishi, A Wei

**Affiliations:** 1 Department of Clinical Haematology, The Alfred Hospital and Monash University, Melbourne, Victoria, Australia; 2 Department of Haematology, St Vincent's Hospital, East Melbourne, Victoria, Australia; 3 Department of Haematology, Westmead Hospital, Westmead, New South Wales, Australia; 4 Department of Haematology, Royal Brisbane Hospital, Herston, Queensland, Australia; 5 Department of Haematology, The Royal Melbourne Hospital, Melbourne, Victoria, Australia; 6 Department of Haematology, Canberra Hospital, Garran, Australian Capital Territory, Australia; 7 Department of Haematology, Cairns and Hinterland Hospital and Health Service, Cairns, Queensland, Australia

Salvage chemotherapy for relapsed/refractory FLT3-ITD-mutant acute myeloid leukemia (AML) is associated with a low complete remission (CR) rate (20–26%), despite the sequential use of a FLT3 inhibitor.^1^ FLT3 ligand rises precipitously after chemotherapy administration in advanced AML and is thought to have a detrimental effect on the activity of FLT3 inhibitors.^1^ Most studies involving FLT3 inhibitors deliver the tyrosine kinase inhibitor (TKI) concomitantly with, or sequentially after, chemotherapy. This followed prior assertions that FLT3 inhibitors could induce cell cycle arrest, thus antagonising the effects of cell cycle-dependent chemotherapy.^2^ The clinical effects of FLT3 inhibitor priming in patients with FLT3-ITD AML receiving chemotherapy has not been previously reported. Pre-clinical studies by Taylor *et al.*^3^ proposed that FLT3 inhibitor priming could induce leukemic progenitors into S-phase, thereby sensitising FLT3-ITD-mutant AML to subsequent chemotherapy. Administration of FLT3 inhibitors before chemotherapy may avoid the neutralising effects of rising FLT3 ligand levels after chemotherapy.^1^ Furthermore, a non-cytotoxic pre-phase may attenuate the risks associated with tumour lysis syndrome in patients with severe baseline hyperleukocytosis. We therefore report the outcome of 10 patients with relapsed or refractory FLT3-ITD AML treated with the multikinase (including FLT3) inhibitor sorafenib (400 mg b.i.d.) for 7 days as pre-phase, followed by salvage chemotherapy with FLAG–Amsa (fludarabine 30 mg/m^2^ days 1–5, cytarabine 2 g/m^2^ days 1–5, G-CSF 300 μg subcutaneously days 0–6 and amsacrine 100 mg/m^2^ days 1–3). Patients received sorafenib from their treating physicians in an off-label manner. The schedule allowed the effects of sorafenib priming to be assessed without the confounding effects of further TKI prior to response evaluation. Restriction of sorafenib to 7 days during salvage was also a pragmatic one to minimise costs related to hospital-funded drug provision. Sorafenib is known to be metabolised by CYP3A4 to sorafenib N-oxide, which has active potency against FLT3-ITD.^4^ Azoles were therefore avoided during the sorafenib pre-phase. Among the 10 patients treated, CR or CR with incomplete blood count recovery (CRi) was achieved in 50% (Table 1). Sorafenib was highly effective in rapidly suppressing hyperleukocytosis in two patients (#6 and #9) with baseline peripheral blood white cell counts falling from 176 and 184 × 10^9^/l on day 1, to 0.9 and 2.1 × 10^9^/l on day 7, respectively (Table 1). Three patients who achieved CR/CRi remain alive after 19+ (#1), 14+ (#2) and 2 (#5) months. In two patients, serum FLT3 ligand levels were obtained. Plasma FLT3 ligand levels did not rise above 70 pg/ml in either patient during the first week of sorafenib (not shown). These results suggest that FLT3 inhibitors given as pre-phase before chemotherapy does not impede the clinical response to salvage therapy in patients with relapsed/refractory FLT3-ITD-mutant AML while delivering rapid cytoreductions in those affected by severe hyperleukocytosis before chemotherapy. Response durations were short in three of the five patients, suggesting the need for additional post-remission strategies. Salvage therapy with sorafenib–FLAG–Amsa, involving only 7 days of sorafenib exposure before chemotherapy, was an economically prudent, well-tolerated and efficacious regimen in relapsed/refractory FLT3-ITD AML.

## Figures and Tables

**Table 1:** Patient characteristics, response and outcome

*Pt*	*Age*	*CG*	*Prior therapy*	*Sorafenib day and WCC x 10*^*9*^*/l*	*Marrow response day 28 post sorafenib–FLAG–Amsa*	*Subsequent therapy*	*OS (months)*
1	62	N	7+3	D1= n/a D7=3.0	CRi	AlloSCT	19+
2	40	N	HiDAC-3, AlloHSCT	D1= n/a D7= 2.6	CRi	DLI, sorafenib	14+
3	17	N	7+3	D1=0.9 D7=0.9	CRi	DUCBT	5
4	44	N	7+3	D1=0.3 D7=0.2	CRi	Nil	4
5	55	+4	7+3, HiDAC-1	D1=1.3 D7=6.4	CR	Sorafenib–FLAG–Amsa	2+
6	46	+8	HiDAC-3	D1=184 D7=2.1	Resistant	AlloSCT, sorafenib	8
7	24	+8	HiDAC-3, AlloHSCT	D1=0.6 D7=0.5	Resistant	DLI, melphalan, clinical trials	7
8	25	N	7+3	D1=176 D7=0.9	Resistant	Hydroxyurea Thioguinine, sorafenib	6
9	34	+8	7+3	D1=27.6 D7=4.9	Resistant	Nil	5
10	64	N	ICE, 5+2	D1=22 D7=2.8	Resistant	Nil	2

Abbreviations: alloSCT, allogeneic stem cell transplant; CG, cytogenetics; CR, complete remission; CRi, complete remission with incomplete blood count recovery; DLI, donor lymphocyte infusion; DUCBT, double unrelated cord blood transplant; FLAG–Amsa, see Fong *et al.*^5^; HiDAC-3, cytarabine 3 g/m^2^ bd. days 1, 3, 5, 7+idarubicin 12 mg/m^2^ days 1–2; ICE, idarubicin 9 mg/m^2^ days 1–3+cytarabine 3 g/m^2^ bd days 1,3,5,7+etoposide 75 mg/m^2^ days 1–7; 5+2, cytarabine 100 mg/m^2^ days 1–5+idarubicin 12 mg/m^2^ days 1–2; N, normal; n/a, result not available; Pt, patient; WCC, white cell count; 7+3, cytarabine 100 mg/m^2^ days 1–7+idarubicin 12 mg/m^2^ days 1–3.
